# Transcriptome Analyses Identify a Metabolic Gene Signature Indicative of Antitumor Immunosuppression of EGFR Wild Type Lung Cancers With Low PD-L1 Expression

**DOI:** 10.3389/fonc.2021.643503

**Published:** 2021-09-14

**Authors:** Min Wang, Jie Zhu, Fang Zhao, Jiani Xiao

**Affiliations:** ^1^Department of Respiratory and Geriatrics, Chongqing Public Health Medical Center, Chongqing, China; ^2^Department of Respiratory and Critical Care Medicine, West China Hospital, Sichuan University, Chengdu, China; ^3^Department of Intensive Care Unit, The People’s Hospital of Tongliang District, Chongqing, China; ^4^Department of Pediatric Dentistry, West China Hospital of Stomatology, Sichuan University, Chengdu, China

**Keywords:** epidermal growth factor receptor (EGFR) wild-type, low expression of programmed death-ligand 1 (PD-L1), lung adenocarcinoma, lung squamous carcinoma, The Cancer Genome Atlas Program (TCGA), GEO

## Abstract

**Purpose:**

With the development and application of targeted therapies like tyrosine kinase inhibitors (TKIs) and immune checkpoint inhibitors (ICIs), non-small cell lung cancer (NSCLC) patients have achieved remarkable survival benefits in recent years. However, epidermal growth factor receptor (EGFR) wild-type and low expression of programmed death-ligand 1 (PD-L1) NSCLCs remain unmanageable. Few treatments for these patients exist, and more side effects with combination therapies have been observed. We intended to generate a metabolic gene signature that could successfully identify high-risk patients and reveal its underlying molecular immunology characteristics.

**Methods:**

By identifying the bottom 50% PD-L1 expression level as PD-L1 low expression and removing EGFR mutant samples, a total of 640 lung adenocarcinoma (LUAD) and lung squamous carcinoma (LUSC) tumor samples and 93 adjacent non-tumor samples were finally extracted from The Cancer Genome Atlas (TCGA). We identified differentially expressed metabolic genes (DEMGs) by R package limma and the prognostic genes by Univariate Cox proportional hazards regression analyses. The intersect genes between DEMGs and prognostic genes were put into the least absolute shrinkage and selection operator (LASSO) penalty Cox regression analysis. The metabolic gene signature contained 18 metabolic genes generated and successfully stratified LUAD and LUSC patients into the high-risk and low-risk groups, which was also validated by the Gene Expression Omnibus (GEO) database. Its accuracy was proved by the time-dependent Receiver Operating Characteristic (ROC) curve, Principal Components Analysis (PCA), and nomogram. Furthermore, the Single-sample Gene Set Enrichment Analysis (ssGSEA) and diverse acknowledged methods include XCELL, TIMER, QUANTISEQ, MCPcounter, EPIC, CIBERSORT-ABS, and CIBERSORT revealed its underlying antitumor immunosuppressive status. Besides, its relationship with somatic copy number alterations (SCNAs) and tumor mutational burden (TMB) was also discussed.

**Results:**

It is noteworthy that metabolism reprogramming is associated with the survival of the double-negative LUAD and LUSC patients. The SCNAs and TMB of critical metabolic genes can inhibit the antitumor immune process, which might be a promising therapeutic target.

## Introduction

Tyrosine kinase inhibitors (TKIs), as a milestone treatment against lung cancer, have demonstrated remarkable therapeutic effects in NSCLC. TKIs reversibly binds to the intracellular tyrosine kinase domain of the epidermal growth factor receptor (EGFR) by competing with ATP and inhibit activation of downstream signaling (1). Although in EGFR mutation-positive patients, erlotinib, gefitinib, and afatinib have achieved better progression-free survival (PFS) and overall survival (OS), most patients inevitably acquired resistance to TKIs within 12 months (2). Moreover, they are not always beneficial for EGFR mutation-negative patients. Programmed death-ligand 1 (PD-L1) is expressed on multiple malignant tissues and up-regulated within the tumor microenvironment, resulting in T-cell immunity resistance (3). Antibodies of PD-L1 can restore T cell function and enhance antitumor immunity (4).

For patients with EGFR mutation-negative and overexpressing PD-L1, T cell-based immunotherapies, which have been called immune checkpoint inhibitors (ICIs), were used as a choice because of their remarkable clinical response (5, 6). Immunotherapy is the first-line treatment of advanced-stage NSCLC patients harboring EGFR/ALK (ALK receptor tyrosine kinase) wild type with PD-L1 expression ≥ 50% and second-line treatment when PD-L1 expression ranges between 1 and 50% (7). When the expression of PD-L1 ranges between 1-50%, ICIs is still a second-line treatment option along with chemotherapy (5, 6). However, low expression of PD-L1, EGFR wild-type NSCLCs showed less therapeutic benefit and more adverse events. Therapies combined PD-L1 blockade and chemotherapy have achieved modest response rates but at the expense of more adverse effects (8–10). Considering that the carcinogenic mechanism and molecular basis of EGFR wild-type and low expression of PD-L1 NSCLC remain elusive, exploring an optimal treatment regimen is still ambiguous.

Accumulating evidence has suggested that metabolic reprogramming contributes to tumorigenesis and impacts the tumor microenvironment (11). Metabolic gene alterations and their prognostic roles have been observed in several tumor types like thyroid cancer, neuroblastoma, and melanoma, which indicate that altered metabolic genome may serve as novel tumor targets for therapies (12–14). However, it remains unknown whether metabolism-associated genes could mediate a key mechanism in EGFR wild-type and low expression of PD-L1 NSCLC.

We conducted comprehensive analyses integrating multiple sources in the present work, including gene transcriptome, somatic copy number alterations (SCNAs), mutations, and clinical parameters to uncover the impacts of metabolic genes in the double-negative (EGFR wild-type and low expression of PD-L1) LUAD and LUSC patients. The multiple platforms utilized in this study contain the Cancer Genome Atlas (TCGA), the Gene Expression Omnibus (GEO) database, and website resources like the cBioPortal platform and TIMER. The critical prognostic metabolic genes and a risk predict signature was generated to discuss specific immune status further.

## Materials and Methods

### Sample Datasets and Data Availability

Publicly gene expression and mutation format files of lung adenocarcinoma (LUAD) and lung squamous carcinoma (LUSC) with corresponding clinical data were downloaded from The Cancer Genome Atlas (TCGA) on 1 September 2020, which comprised of 904 EGFR wild type samples and 93 adjacent non-tumor samples. The definition of TMB refers to the number of somatic mutations, coding mutations, base replacement mutations, and insertion mutations per megabase in the genome. We calculated TMB as the number of all mutations/exon length (38 million) for each sample. By identifying the bottom 50% PD-L1 expression level as PD-L1 low expression and removing EGFR mutant samples, 640 tumor samples were finally extracted. The LUAD and LUSC expression dataset in Gene Expression Omnibus (GEO) were included as the validation cohorts. GSE3141 contained gene expression profile of 111 primary lung tumor samples and corresponding prognostic data. GSE14814 included 133 NSCLC samples with clinical data and microarray data.

### Gene Expression Data Analysis

The metabolism-related genes were obtained from the Molecular Signatures Database (MSigDB) of the gene set enrichment analysis (GSEA) website. 944 genes in the KEGG (Kyoto Encyclopedia of Genes and Genomes) gene sets that correlated with the metabolism pathway were identified and extracted. Differentially expressed metabolic genes (DEMGs) between tumor and normal tissues were targeted by using the R package limma. FDR > 0.05 and |log2FoldChange| > 1 were defined as the thresholds for screening DEMGs to identify upregulated and downregulated genes. We performed the Univariate Cox proportional hazards regression analyses to determine the prognostic genes associated with overall survival (OS). The intersect genes between DEMGs and prognostic genes were considered to put into the least absolute shrinkage and selection operator (LASSO) penalty Cox regression analysis.

### Construction of the Prognostic Metabolic Signatures

The LASSO penalty Cox regression analysis was conducted on these intersect genes and to generate a metabolic gene signature. By calculating each subject’s cox regression coefficients, patients were sub-grouped to the high- and low-risk depending on their median value of signature scores. The same signature formula was applied to the GEO validation cohorts. The Kaplan–Meier method was achieved by the survival and survivalROC packages. The time-dependent Receiver Operating Characteristic (ROC) curve was used to predict clinical characteristics’ accuracy and signature. The R package rms generated the nomograms. The calibration curve showed the calibration of the nomogram between the predicted risk and observed outcomes, representing the predicted and actual 3-Year overall survival (OS).

### Gene Enrichment Analysis and Tumor-Infiltrating Cells Analysis

Single-sample Gene Set Enrichment Analysis (ssGSEA) is an extension of Gene Set Enrichment Analysis (GSEA), which calculates separate enrichment scores for each pairing of a sample and gene set. The ssGSEA scores for most immune cell populations were obtained using the gene sets from Angelova et al. (15). A total of 29 gene sets representing distinct immune cell populations were obtained: activated dendritic cells (aDCs), antigen-presenting cell (APC) co-inhibition, APC co-stimulation, B cells, CC chemokine receptor (CCR), CD8+ T cells, Check-point, Cytolytic activity, dendritic cells (DCs), human leukocyte antigen (HLA), interdigitating dendritic cells (iDCs), Inflammation-promoting, Macrophages, Mast cells, MHC class I, Neutrophils, NK cells, Parainflammation, plasmacytoid dendritic cells (pDCs), T cell-co-inhibition, T cell co-stimulation, T helper cells, follicular helper T cells (Tfh), Th1 cells, Th2 cells, tumor-infiltrating lymphocytes (TIL), regulatory T cells (Treg), Type I IFN Response, Type II IFN Response (16, 17).

The current acknowledged methods such as XCELL (18), TIMER (19), QUANTISEQ (20), MCPcounter (21), EPIC (22), CIBERSORT-ABS (23), and CIBERSORT (24) were united to reveal the immunologic characteristics between groups. Diverse immune infiltrating cells were estimated by Spearman correlation analysis and Wilcoxon signed-rank test with risk scores and risk groups. The estimation files for the TCGA project calculate the immune infiltration statues were download from the TIMER website (http://timer.comp-genomics.org). The R packages ggplot2, ggtext, scales, and limma were used in this procedure.

### The Cancer Immunome Atlas

The Cancer Immunome Atlas (TCIA) is an online searchable database that enables researchers to develop and test hypotheses on the impact of cancer genome on tumor microenvironment and immune characteristics, particularly concerning ICIs treatment response (25). The immunophenotypes of 20 solid cancers from TCGA are determined by cellular characterization of the immune infiltrates, showing potential tumor escape mechanisms. Using the machine learning method, tumor immunogenicity is identified, and a scoring scheme defined as immunophenoscore is generated. The immunophenoscore can be used as a favorable predictor of response to anti-CTLA-4 and anti-PD-1 antibodies, which were validated in two independent cohorts. A higher immunophenoscore indicates a better prognosis and better response to immunotherapy (26). Through TCIA, we obtained the immunophenoscore of TCGA-LUAD and TCGA-LUSC and the expression levels of CTLA-4 and PD-1. Patients were re-grouped according to the expression of CTLA-4 and PD-1, and then the immunophenoscore of the high- and low-risk patients were compared for responses to ICIs treatment.

### Function Annotation

By taking the median risk value as the threshold, samples were divided into the high- and low-risk groups. General differentially expressed genes (DEGs) were screened between these two risk levels and were put into the Gene Ontology (GO) function annotation and Kyoto Encyclopedia of Genes and Genomes (KEGG) pathway analysis by R package GOplot.

### Statistical Analysis

The student’s t-test compared gene expression data between tumor samples and adjacent non-tumor samples. The Chi-square test for parametric distributions or the Wilcoxon test for nonparametric distributions was used for differences in proportions. All statistical analyses were performed with R software (Version 3.6.3) and Graphpad (Version 8.0.2). P-value < 0.05 was considered statistically significant if there are not specially mentioned.

## Results

### Identification and Validation of the Prognostic DEMGs Signature in the TCGA Cohort

**Figure 1:**
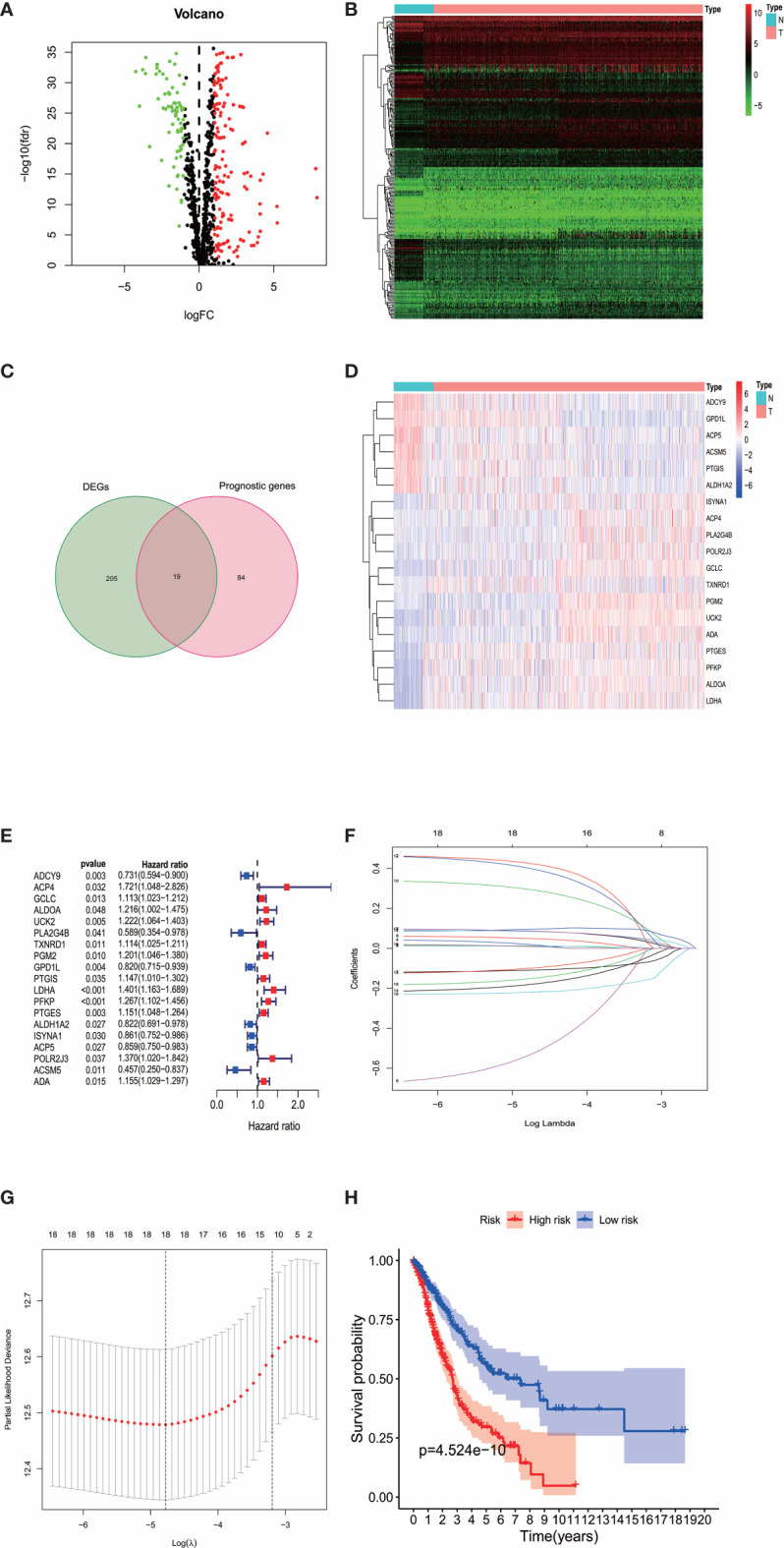
**(A)** The volplot of DEMGs between tumor and normal tissues. The red plots represent up-regulated genes and the green plots represent down-regulated genes. **(B)** The heatmap of DEMGs between tumor and normal tissues. N represents normal tissues and T represents tumor tissues. **(C)** The intersection of DEMGs and the prognostic genes were generated. A total of 19 intersection genes were identified. **(D)** The heatmap of the 19 intersection genes. **(E)** The 19 intersection genes that related to OS were extracted by univariate Cox regression analysis. **(F, G)** The LASSO Cox regression analysis were performed with the optimal value of λ. **(H)** The survival analysis revealed the high-risk patients were associated with poor prognosis.

In 944 metabolism-related genes, 224 DEMGs were identified between tumor tissues and adjacent nontumorous tissues (73 down-regulated and 151 up-regulated, **Figures 1A, B**), of which 103 metabolic genes related to OS were extracted by univariate Cox regression analysis (**Figure 1C**). After cross-matching, 19 DEMGs correlated with OS were finally generated and were put in LASSO Cox regression analysis to establish a prognostic signature (**Figures 1D, E**). Based on the optimal value of λ (**Figures 1F, G**), a signature comprised of 18 DEMGs was constructed as follows: risk score = ADCY9*-0.1096 + ACP4*0.4257 + GCLC*0.0124 + ALDOA*0.0196 + UCK2*0.0106 + PLA2G4B*-0.5342 + TXNRD1*0.0865 + PGM2*0.0496 + PTGIS*0.2946 + LDHA*0.0949 + PFKP*0.0838 + PTGES*0.0865 + ALDH1A2*-0.1811 + ISYNA1*-0.1000 + ACP5*-0.1516 + POLR2J3*0.4008 + ACSM5*-0.2192 + ADA*0.0094.

**Figure 2:**
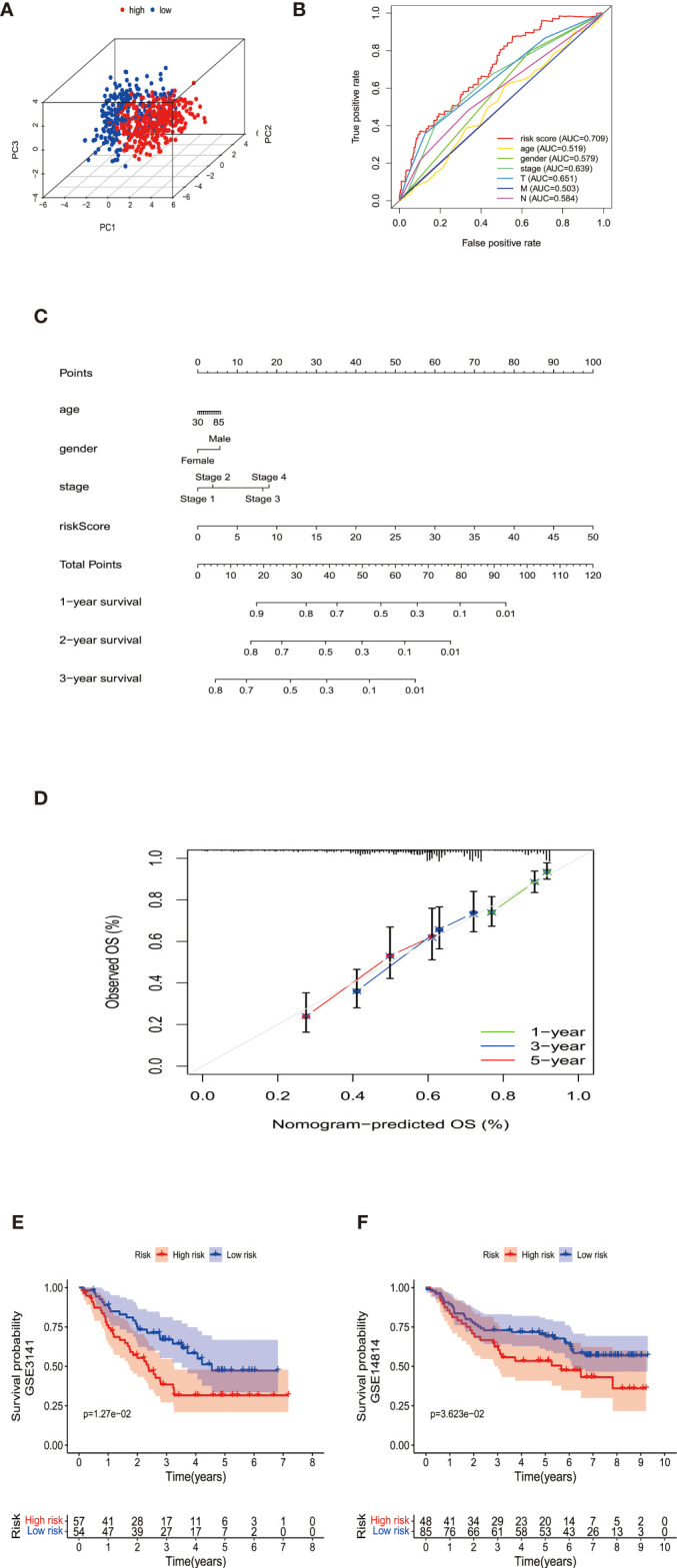
**(A)** The PCA plots showed the metabolic gene signature could successfully distinguish the double-negative LUAD and LUSC patients. **(B)** The ROC curve analyses indicated that the AUC value (0.709) of the DEMGs signature was higher than all the other risk factors. **(C)** The nomogram was built to incorporate the clinical parameters and risk score to predict 1-,2-, and 3-year OS. **(D)** The nomogram’s 1-,3-, and 5-year calibrate curve revealed that the predicted OS was very close to the actual OS, indicating high accuracy. **(E, F)** The Kaplan-Meier plot of GSE3141 and GSE14814 showed the patients in the high-risk group had a significantly poor survival time than those in the low-risk group.

According to the median cut-off value of risk score, the patients were divided into the high- and low-risk groups. The higher risk score was associated with worse OS in survival analysis (**Figure 1H**). We used Principal Component Analysis (PCA) to evaluate the prognostic signature’s effectiveness, which showed significantly different distribution patterns and suggested that the DEMGs signature can distinguish the high- and low-risk patients effectively (**Figure 2A**). The ROC curve analyses indicated that the AUC value of the DEMGs signature was higher than all the other risk factors (**Figure 2B**), reaching 0.709. A nomogram was also built to incorporate the clinical parameters and risk score to predict 3-year OS. The nomogram’s 1-, 3- and 5-year calibrate curve revealed that the predicted OS was very close to the actual OS (**Figures 2C, D**), indicating high accuracy.

The DEMGs signature was tested in cohorts GSE3141 and GSE14814 for validation. We took the same formula to predict patients’ survival, of which the Kaplan-Meier plot showed the patients in the high-risk group had a significantly poor survival time than those in the low-risk group (**Figures 2E, F**).

### Associations Between the Prognostic Signature and Clinicopathologic Features

**Figure 3:**
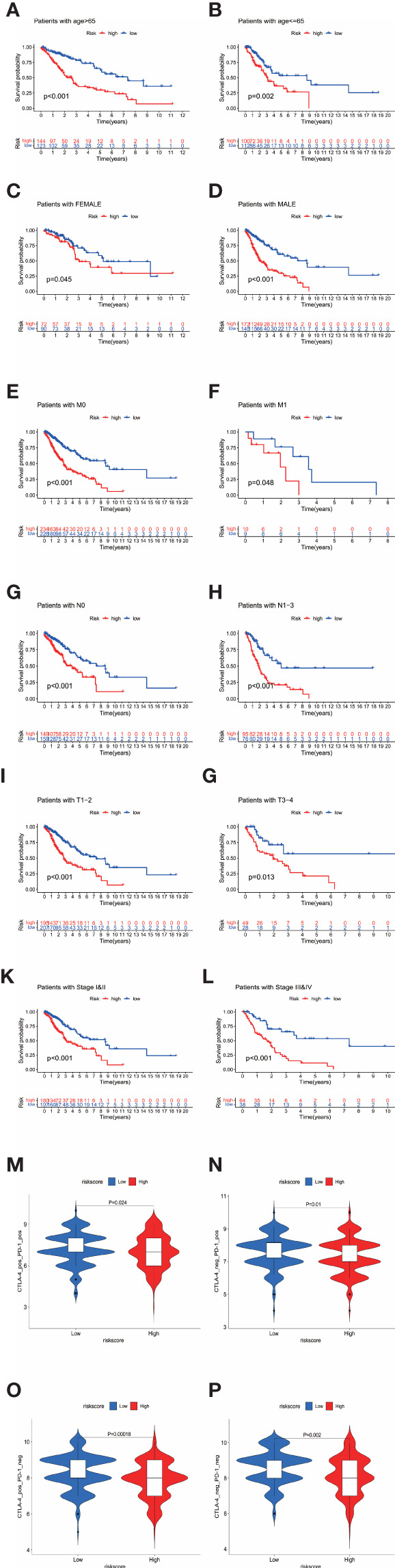
**(A–L)** The prognostic signature well divided the high- and low- risk patients for survival in diverse clinical parameters (age, gender, M stage, N stage, T stage, and pathological stage). **(M–P)** The low-risk patients showed higher immunophenoscores in all four subgroups, CTLA-4 positive PD-1 positive, CTLA-4 negative PD-1 positive, CTLA-4 positive PD-1 negative, and CTLA-4 negative PD-1 negative, suggesting a better response to immunotherapies regardless of the expression of CTLA-4 and PD-1.

As shown in **Figures 3A–L**, we further tested the relationship between the signature and pathological parameters. In younger or older patients, female and male patients, early- or late-stage patients, the signature still showed the prognostic ability in the high- and low-risk groups.

### GO Function Annotation for the Prognostic Signature

**Figure 4:**
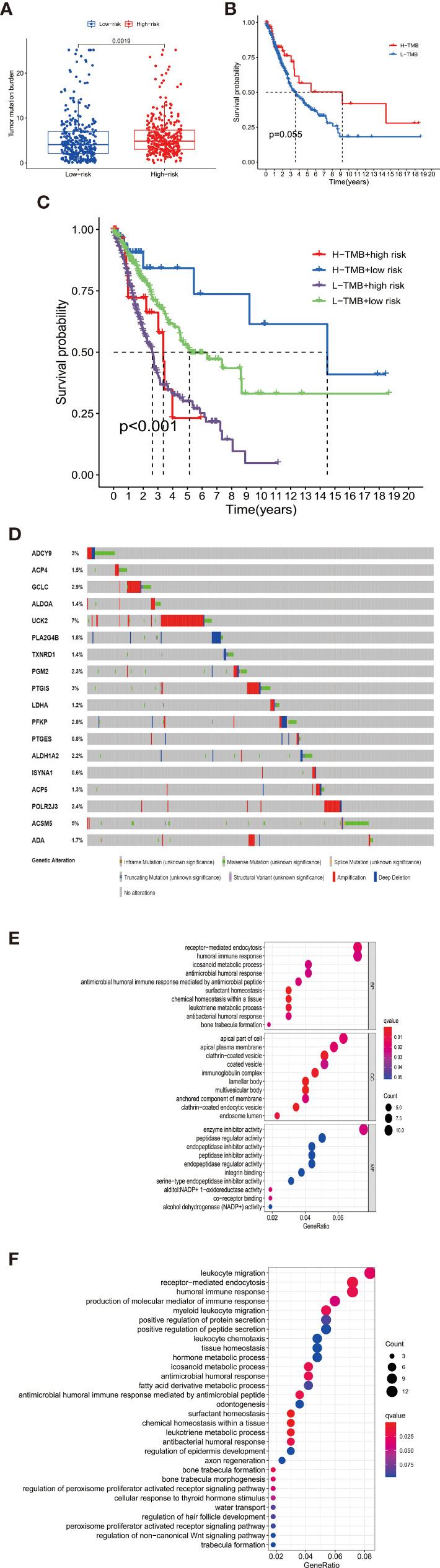
**(A)** The high-risk group had a higher TMB level than the low-risk group, indicating a reverse TMB tendency compared with immunology status in high-risk patients. **(B)** There was no difference in survival between the high-TMB and low-TMB patients. **(C)** When the signature groups were added to TMB, the prognosis of patients in different subgroups showed distent survival differences. Patients with high-TMB and low-risk had best prognosis among all the groups. **(D)** The 18 signature genes showed varying levels of mutation in TCGA-LUAD and LUSC samples. The mutation rates of UCK2 and ACSM5 were significantly higher than that of other genes. **(E, F)** The barplot of Go and KEGG functional enrichment analyses. BP indicated biological process; CC indicated cellular component; MF indicated molecular function.

Patients were divided into the high- and low-risk groups depending on the median value of risk scores. The general DEGs between two risk levels were put into GO and KEGG functional annotation (**Figures 4E, F**), which revealed that diverse immunology processes pathways like humoral immune response, leukocyte migration, leukocyte chemotaxis, and leukotriene metabolic process were involved.

### Genetic Alterations of the Genes in the Prognostic Signature

The mutation data of 586 LUAD samples and 501 LUSC samples are available in the cBioPortal platform (http://www.cbioportal.org/). As shown in **Figure 4D**, the 18 signature genes showed varying levels of mutation in TCGA-LUAD and LUSC samples. The mutation rates of UCK2 and ACSM5 were significantly higher than that of other genes.

**Figure 5:**
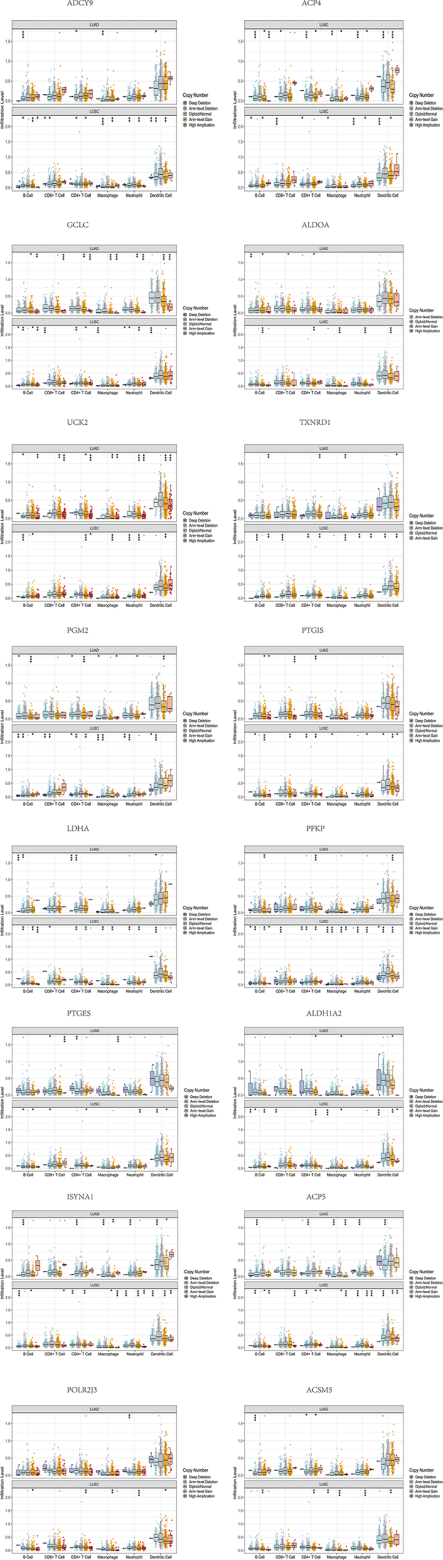
The differential SCNAs categories of the signature genes statistically changed the immune cell infiltration levels, including B cell, CD8+ T cell, CD4+ T cell, macrophage, neutrophil, and dendritic cell infiltration.

Furthermore, we investigated the impacts of the 18 signature genes on immune infiltrations *via* the TIMER database (https://cistrome.shinyapps.io/timer/), a comprehensive resource for systematical analysis of immune infiltrates across diverse cancer types (19). We evaluated the relationship between somatic copy number alterations (SCNAs) of 14 signature genes and immune cell infiltration. The SCNAs includes deep deletion (-2), arm-level deletion (-1), diploid/normal (0), arm-level gain (1), and high amplification (2). The immune infiltration level in each category was compared with the diploid/normal level. A p-value < 0.05 was considered significant, and a two-sided Wilcoxon rank-sum test was used between different categories. As shown in **Figure 5**, differential SCNAs categories statistically changed the immune cell infiltration levels, including B cell, CD8+ T cell, CD4+ T cell, macrophage, neutrophil, and dendritic cell infiltration.

### Immune Profile Analysis

**Figure 6:**
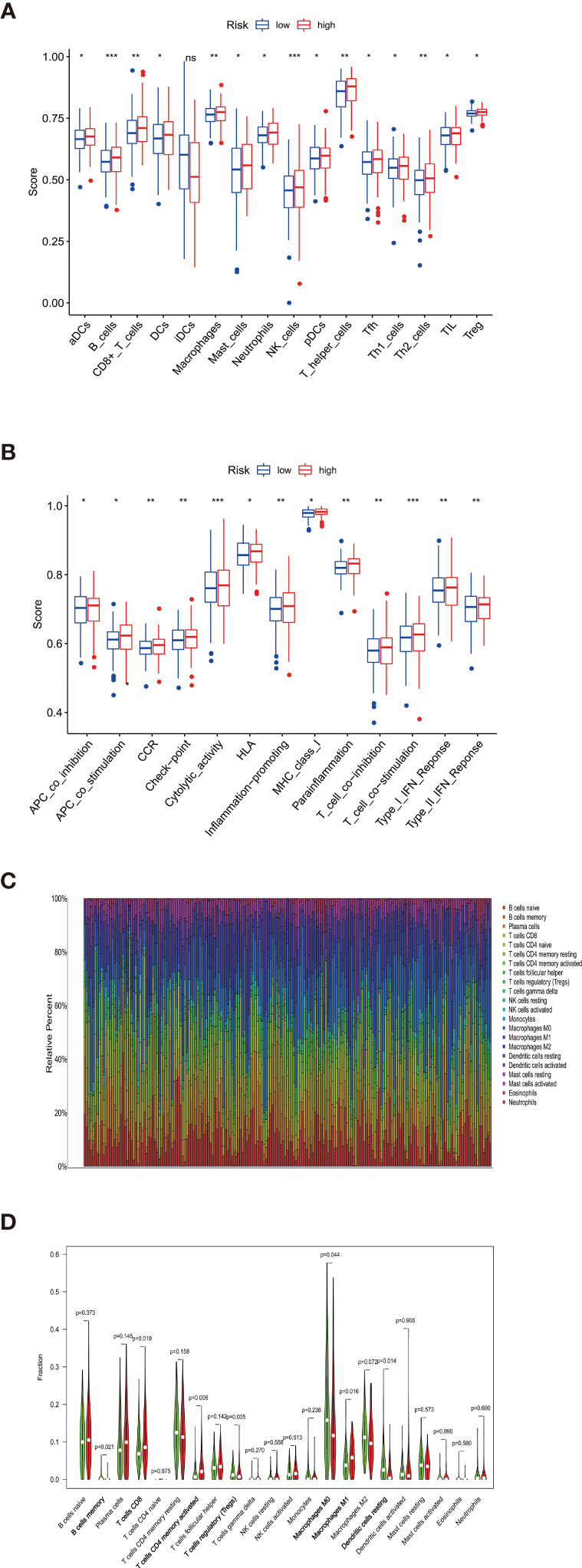
**(A)** aDCs, B cells, CD8+ T cells, DCs, Macrophages, Mast cells, Neutrophils, NK cells, pDCs, T helper cells, Tfh, Th1 cells, Th2 cells, TIL, and Treg were associated with lower ssGSEA score in the high-risk group **(B)** APC co-inhibition, APC co-stimulation, CCR, Check−point, Cytolytic activity, HLA, Inflammation−promoting, MHC class I, Parainflammation, T cell co−inhibition, T cell co−stimulation, Type I IFN Reponse, and Type II IFN Response gained lower ssGSEA score in the high-risk group. **(C)** Taking the median TMB value as a cutoff, the relative expression of 22 tumor-infiltrating immune cells in the low- and high-TMB samples was determined. **(D)** B cells memory, T cells CD8, T cells CD4 memory activated, Tregs, Macrophages M0, Macrophages M1, and Dendritic cells resting showed infiltration differences between the low- and high-TMB samples. *P < 0.05, **P < 0.01, ***P < 0.001 and ns, no statistical significance.

We characterized the immunology profile of EGFR wild type lung cancer samples with low PD-L1 expression by ssGSEA. In 29 immune gene sets, aDCs, B cells, CD8+ T cells, DCs, Macrophages, Mast cells, Neutrophils, NK cells, pDCs, T helper cells, Tfh, Th1 cells, Th2 cells, TIL, and Treg were associated with lower ssGSEA score in the high-risk group (**Figure 6A**). Similarly, APC co-inhibition, APC co-stimulation, CCR, Check−point, Cytolytic activity, HLA, Inflammation−promoting, MHC class I, Parainflammation, T cell co−inhibition, T cell co−stimulation, Type I IFN ReSponse, and Type II IFN Response gained lower ssGSEA score in the high-risk group (**Figure 6B**). The general immune cell types and immune response were significantly lower in high-risk double-negative (with EGFR wild and low PD-L1 expression) LUAD and LUSC, suggesting immunosuppression statuses in the high-risk patients.

**Figure 7:**
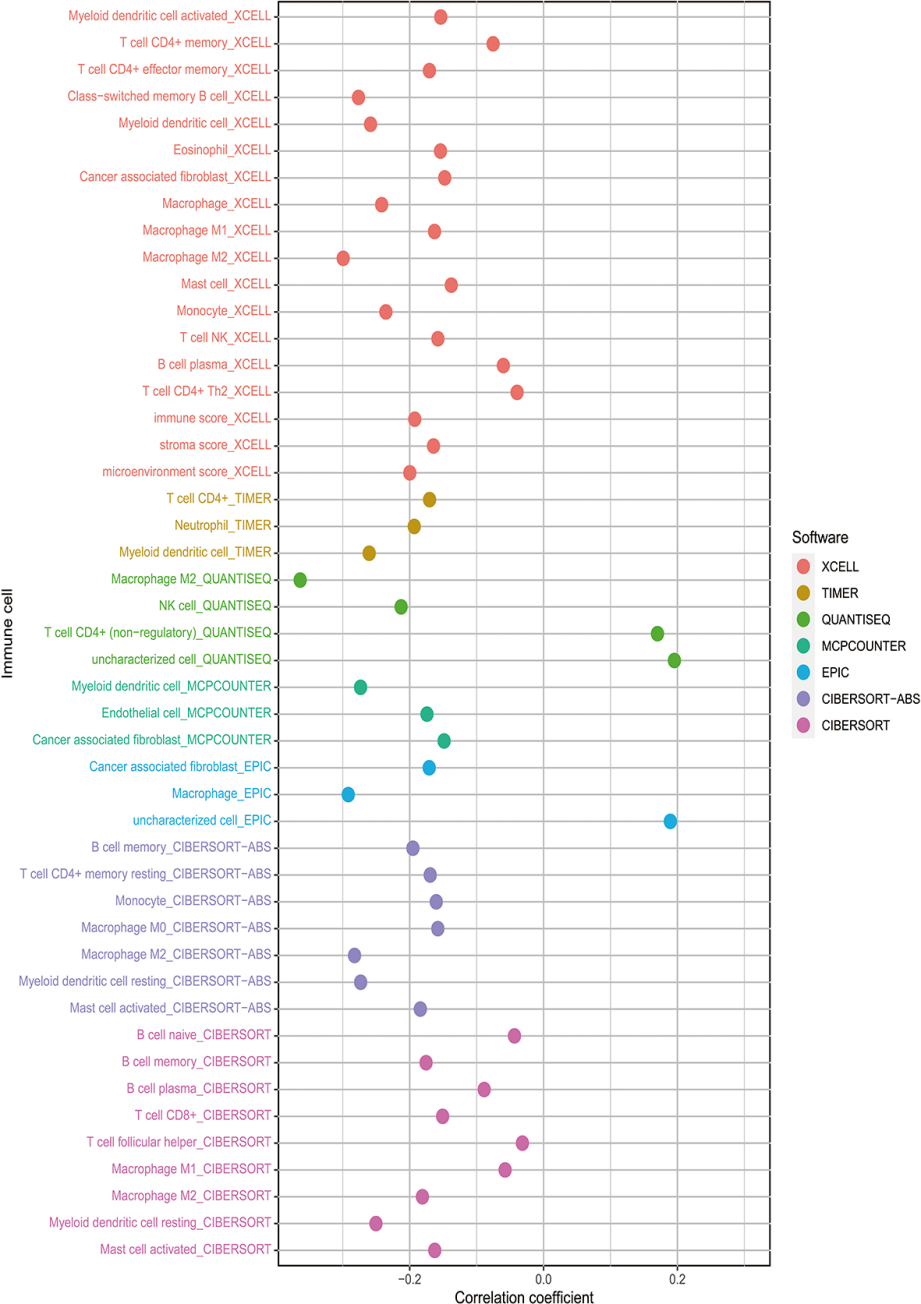
The risk score was negatively associated with most tumor-infiltrating immune cells shown by Spearman correlation analysis in current acknowledged methods such as XCELL, TIMER, QUANTISEQ, MCPcounter, EPIC, CIBERSORT-ABS, and CIBERSORT.

**Figure 8:**
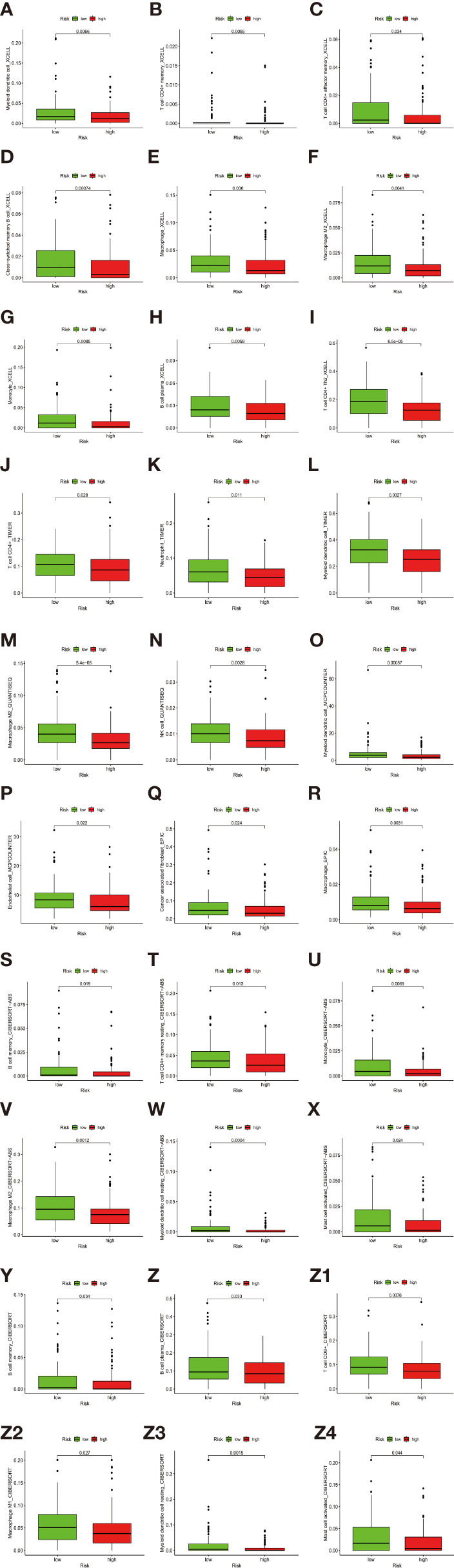
**(A–Z4)** The Wilcoxon signed-rank test revealed the lower levels of extensive immune infiltration cells such as T cell CD4+ central memory, T cell CD4+ Th2, T cell CD8+, T cell CD8+ naive, myeloid dendritic cell, and macrophage.

Besides, The Spearman correlation analysis also indicated a negative relationship between risk score and multiple immune infiltration cells. As shown in **Figure 7**, most recognized immune infiltrating cells were negatively correlated with risk scores, including diverse T cells, B cells, and Macrophage. The Wilcoxon signed-rank test also confirmed the analysis of ssGSEA. In **Figure 8**, immunosuppression statuses in the high-risk patients were revealed by the lower levels of extensive immune infiltration cells such as T cell CD4+ central memory, T cell CD4+ Th2, T cell CD8+, T cell CD8+ naive, myeloid dendritic cell, and macrophage.

### The Immunologic Landscape of TMB and Its Correlation With the Prognostic Signature

The definition of TMB is the number of mutations per DNA megabases (Mb). TMB has been reported to closely influence the response to ICIs (27–29) because the highly mutated burdens are associated with abundant neoantigens and susceptibility to immune cells (29, 30).

Considering the extensive association between TMB and the tumor immune microenvironment, we further tested the immunologic landscape of TMB and its correlation with the prognostic signature. As shown in **Figure 4A**, the high-risk group had a higher TMB level than the low-risk group, indicating a reverse TMB tendency compared with immunology status in the high-risk patients.

When TMB alone was used as a prognostic indicator, there was no significant difference in survival between the low- and high-TMB groups by taking the median TMB value as cutoff (**Figure 4B**). However, when the signature groups were added to TMB, the prognosis of patients in different subgroups showed distent survival differences (**Figure 4C**). Patients with high-TMB and low-risk had the best prognosis among all the groups.

The proportions of the 22 TILs from each sample were determined by using the R package CIBERSORT. Gene expression profiles were transformed into the proportion of 22 TILs, namely: B cells naive, B cells memory, Plasma cells, T cells CD8, T cells CD4 naive, T cells CD4 memory resting, T cells CD4 memory activated, T cells follicular helper, T cells regulatory (Tregs), T cells gamma delta, NK cells resting, NK cells activated, Monocytes, Macrophages M0, Macrophages M1, Macrophages M2, Dendritic cells resting, Dendritic cells activated, Mast cells resting, Mast cells activated, Eosinophils, and Neutrophils (**Figure 6C**). Significant results (P < 0.05) were selected for subsequent analysis. Taking the median TMB value as a cutoff, the relative expression of 22 tumor-infiltrating immune cells in the low- and high-TMB samples were determined (**Figure 6D**). B cells memory, T cells CD8, T cells CD4 memory activated, Tregs, Macrophages M0, Macrophages M1, and Dendritic cells resting showed infiltration differences relating to the two TMB levels. It is worth mentioning that B cells memory, T cells CD8, T cells CD4 memory activated, and Macrophages M1 play important roles in pro-inflammatory response while showing lower infiltration levels in the high-risk groups, which are consistent with the immunosuppressive status confirmed by ssGSEA.

### Assessment of the Immunophenoscore From The Cancer Immunome Atlas to Predict the Response to ICIs

The negative correlation between risk score and immune infiltration cells was validated above. We further confirmed the relationship between risk score and immunotherapy response by TCIA. TCIA is a web-based database (https://tcia.at/home) that provides information on the cellular composition of tumor infiltrating lymphocytes, informing on response to checkpoint blocker immunotherapies of 20 solid cancers from TCGA. TCIA provided a composite score, the immunophenoscore, to reveal tumor immunologic heterogeneity of 20 valid cancers as an indication of tumor cell type might be susceptible to ICIs treatment. Higher immunophenoscores were positively correlated with better anti-cytotoxic lymphocyte antigen-4 (CTLA-4) and anti-PD-1 treatment response. Depending on the expression of CTLA-4 and PD-1, patients were divided into four groups as CTLA-4 positive PD-1 positive, CTLA-4 negative PD-1 positive, CTLA-4 positive PD-1 negative, and CTLA-4 negative PD-1 negative. The low-risk patients showed higher immunophenoscores in all four subgroups, suggesting a better response to immunotherapies regardless of the expression of CTLA-4 and PD-1 (**Figures 3M–P**).

## Discussion

As LUAD and LUSC patients with EGFR wild type can hardly respond to TKIs treatment, immune checkpoint inhibitors (ICIs) are the optimal first-line choices for them. For patients with double-negative, which means “EGFR wild type and low PD-L1 expression,” combing PD-L1 antibody therapy and chemotherapy can improve outcomes but with more toxicities (31). Thus, new therapeutic strategies are needed for better efficacy.

Our study generated a prognostic DEMGs signature by comprehensive analyses of 640 LUAD and LUSC patients from TCGA, which were also validated in cohorts of the GEO database. Previous studies indicated that patients with EGFR wild type and low PD-L1 expression owned immunosuppressive status associated with less immune checkpoint protein expressions and lymphocyte infiltration (32, 33). Meanwhile, our results showed that the high-risk patients evaluated by the DEMGs signature also expressed a similar immunosuppression status. The immune infiltration cell expressions evaluated by multiple methods were negatively related to risk score, and the pro-inflammatory factors like B cells memory, T cells CD8, T cells CD4 memory activated, and Macrophages M1 showed lower infiltration levels in the high-risk group. CD4+ memory T cells and B cells are localized and enriched in tertiary lymphoid structures, showing benefits on tumors’ prognosis (34, 35). Memory B cells function in both naïve and memory T cell responses as antigen-presenting cells, thus inducing an antitumor immune response (36). Besides, we found that although all the 18 signature genes had relatively stable mutation status in LUAD and LUSC, diverse forms of SCNAs in these genes could significantly inhibit immune infiltration in LUAD and LUSC. These may explain the underlying mechanism of antitumor immune deficiency in the double-negative high-risk patients.

Notably, studies reported that TMB could be used as a genomic biomarker that predicts favorable responses to ICIs. In a meta-analysis, patients with higher TMB who underwent ICIs had a better OS and PFS than those who received chemotherapy alone. While in patients with lower TMB, such ICI benefits were not statistically significant (37). In advanced NSCLC patients, this association between high-level TMB and clinical benefit of ICIs was also observed (38). Moreover, higher TMB has been proved to be associated with a lower proportion of antitumor immune cells like macrophages M1, CD8 T cells, and B cells in papillary thyroid carcinoma (39), which was also confirmed by our result. Extensive analysis showed that different TMB levels in the double-negative LUAD and LUSC patients were correlated to variable immune infiltration cells. Combining the signature with TMB, poor prognostic patients could be better identified, making our signature a new predictive biomarker for ICIs benefits in the double-negative LUAD and LUSC patients.

The biological roles of 18 DEMGs signature genes are not fully investigated. Some of the signature genes are reported to be involved in tumor development. For example, ADCY9 encodes the protein adenyl cyclase type 9, which is related to the activation of intracellular production of cyclic AMP (40). ADCY9 has been reported to involve in the development of tumors. A higher expression level of ADCY9 was found in colon cancer and was an indicator of a bad prognosis (41). GCLC encodes a rate-limited enzyme in the GSH biosynthesis (42). Abnormal expression of GCLC was reported in multiple types of tumors (43, 44). ALDOA is coding genes of Aldolase A that involves in the glycolysis of cancer cells (45). It is related to the poor survival of various types of tumors, including pancreatic cancer, osteosarcoma, and LUSC (46–48). Similarly, TXNRD1 is also highly expressed in diverse malignancies and promotes tumor progression (49, 50). UCK2, a type of rate-limiting enzyme of pyrimidine-nucleotide biosynthesis, is detected to up-regulate in several types of tumors, including neuroblastoma and hepatocellular carcinoma (51, 52). Specifically, a high expression level of UCK2 has also been identified in stage IA lung cancer related to early recurrence, poor first progression survival, and short overall survival (53). In previous studies, PFKP is differentially expressed in glucose metabolic reprogramming in some cancers (54, 55). Namely, PFKP is lowly expressed in seminomas and embryonal carcinomas but highly expressed in human breast tumor cells and lung cancer (56). ISYNA1 is associated with p53-related apoptosis in cancers like lung squamous cell carcinoma, bladder cancer, and pancreatic cancer (57, 58), affecting tumor proliferation and clinical parameters. ACP5 encodes a metalloprotein enzyme that belongs to the acid phosphatase family, up-regulated in breast cancer and LUAD, associated with poor outcomes (59, 60).

In conclusion, metabolic genes play an essential role in risk evaluation of LUAD and LUSC patients with EGFR wild type, low PD-L1 expression. Higher TMB and lower antitumor immune infiltration were found in the high-risk group, indicating poor prognosis in OS. Our related metabolic signature provides an adaptable way to identify potential therapeutic targets, especially SCNAs of the signature genes that could significantly inhibit the immune cells’ infiltration. More biological roles of these metabolic genes are intended to explore for extended clinical significance.

## Data Availability Statement

The original contributions presented in the study are included in the article/supplementary material. Further inquiries can be directed to the corresponding author.

## Author Contributions

JZ, MW, and FZ handled the conceptualization and methodology. JZ and MW handled the software. MW and JX handled the validation. FZ was in charge of the original draft preparation. JZ and JX were responsible for the review and editing. All authors contributed to the article and approved the submitted version.

## Conflict of Interest

The authors declare that the research was conducted in the absence of any commercial or financial relationships that could be construed as a potential conflict of interest.

## Publisher’s Note

All claims expressed in this article are solely those of the authors and do not necessarily represent those of their affiliated organizations, or those of the publisher, the editors and the reviewers. Any product that may be evaluated in this article, or claim that may be made by its manufacturer, is not guaranteed or endorsed by the publisher.

## References

[1] Cataldo VD Gibbons DL Perez-Soler R Quintas-Cardama A. Treatment of non-Small-Cell Lung Cancer With Erlotinib or Gefitinib. N Engl J Med (2011) 364(10):947–55. doi: 10.1056/NEJMct0807960 21388312

[2] Maemondo M Inoue A Kobayashi K Sugawara S Oizumi S Isobe H. Gefitinib or Chemotherapy for Non-Small-Cell Lung Cancer With Mutated EGFR. N Engl J Med (2010) 362(25):2380–8. doi: 10.1056/NEJMoa0909530 20573926

[3] Spranger S Spaapen RM Zha Y Williams J Meng Y Ha TT. Up-Regulation of PD-L1, IDO, and T(regs) in the Melanoma Tumor Microenvironment is Driven by CD8(+) T Cells. Sci Transl Med (2013) 5(200):200ra116. doi: 10.1126/scitranslmed.3006504 PMC4136707 23986400

[4] Lee J Ahn E Kissick HT Ahmed R. Reinvigorating Exhausted T Cells by Blockade of the PD-1 Pathway. For Immunopathol Dis Therap (2015) 6(1-2):7–17. doi: 10.1615/ForumImmunDisTher.2015014188 PMC5341794 28286692

[5] Rittmeyer A Barlesi F Waterkamp D Park K Ciardiello F von Pawel J. Atezolizumab Versus Docetaxel in Patients With Previously Treated non-Small-Cell Lung Cancer (OAK): A Phase 3, Open-Label, Multicentre Randomised Controlled Trial. Lancet (2017) 389(10066):255–65. doi: 10.1016/S0140-6736(16)32517-X PMC6886121 27979383

[6] Fehrenbacher L Spira A Ballinger M Kowanetz M Vansteenkiste J Mazieres J. Atezolizumab Versus Docetaxel for Patients With Previously Treated Non-Small-Cell Lung Cancer (POPLAR): A Multicentre, Open-Label, Phase 2 Randomised Controlled Trial. Lancet (2016) 387(10030):1837–46. doi: 10.1016/S0140-6736(16)00587-0 26970723

[7] Pai-Scherf L Blumenthal GM Li H Subramaniam S Mishra-Kalyani PS He K. FDA Approval Summary: Pembrolizumab for Treatment of Metastatic Non-Small Cell Lung Cancer: First-Line Therapy and Beyond. Oncologist (2017) 22(11):1392–9. doi: 10.1634/theoncologist.2017-0078 PMC5679831 28835513

[8] Langer CJ Gadgeel SM Borghaei H Papadimitrakopoulou VA Patnaik A Powell SF. Carboplatin and Pemetrexed With or Without Pembrolizumab for Advanced, Non-Squamous Non-Small-Cell Lung Cancer: A Randomised, Phase 2 Cohort of the Open-Label KEYNOTE-021 Study. Lancet Oncol (2016) 17(11):1497–508. doi: 10.1016/S1470-2045(16)30498-3 PMC6886237 27745820

[9] Gandhi L Rodriguez-Abreu D Gadgeel S Esteban E Felip E De Angelis F. Pembrolizumab Plus Chemotherapy in Metastatic Non-Small-Cell Lung Cancer. N Engl J Med (2018) 378(22):2078–92. doi: 10.1056/NEJMoa1801005 29658856

[10] Paz-Ares L Vicente D Tafreshi A Robinson A Soto Parra H Mazieres J. A Randomized, Placebo-Controlled Trial of Pembrolizumab Plus Chemotherapy in Patients With Metastatic Squamous NSCLC: Protocol-Specified Final Analysis of KEYNOTE-407. J Thorac Oncol (2020) 15(10):1657–69. doi: 10.1016/j.jtho.2020.06.015 32599071

[11] Pavlova NN Thompson CB. The Emerging Hallmarks of Cancer Metabolism. Cell Metab (2016) 23(1):27–47. doi: 10.1016/j.cmet.2015.12.006 26771115 PMC4715268

[12] Ma B Jiang H Wen D Hu J Han L Liu W. Transcriptome Analyses Identify a Metabolic Gene Signature Indicative of Dedifferentiation of Papillary Thyroid Cancer. J Clin Endocrinol Metab (2019) 104(9):3713–25. doi: 10.1210/jc.2018-02686 30942873

[13] Meng X Feng C Fang E Feng J Zhao X. Combined Analysis of RNA-Sequence and Microarray Data Reveals Effective Metabolism-Based Prognostic Signature for Neuroblastoma. J Cell Mol Med (2020) 24(18):10367–81. doi: 10.1111/jcmm.15650 PMC7521294 32683778

[14] Wan M Zhuang B Dai X Zhang L Zhao F You Y. A New Metabolic Signature Contributes to Disease Progression and Predicts Worse Survival in Melanoma. Bioengineered (2020) 11(1):1099–111. doi: 10.1080/21655979.2020.1822714 PMC8291831 33084485

[15] Angelova M Charoentong P Hackl H Fischer ML Snajder R Krogsdam AM. Characterization of the Immunophenotypes and Antigenomes of Colorectal Cancers Reveals Distinct Tumor Escape Mechanisms and Novel Targets for Immunotherapy. Genome Biol (2015) 16:64. doi: 10.1186/s13059-015-0620-6 25853550 PMC4377852

[16] Zhu J Xiao J Wang M Hu D. Pan-Cancer Molecular Characterization of M(6)A Regulators and Immunogenomic Perspective on the Tumor Microenvironment. Front Oncol (2020) 10:618374. doi: 10.3389/fonc.2020.618374 33585244 PMC7876474

[17] Zhao F Wang M Zhu J. Hypoxia-Related lncRNAs to Build Prognostic Classifier and Reveal the Immune Characteristics of EGFR Wild Type and Low Expression of PD-L1 Squamous and Adenocarcinoma NSCLC. Cancer Med (2021). doi: 10.1002/cam4.4126 PMC8419766 34250747

[18] Aran D. Cell-Type Enrichment Analysis of Bulk Transcriptomes Using Xcell. Methods Mol Biol (2020) 2120:263–76. doi: 10.1007/978-1-0716-0327-7_19 32124326

[19] Li T Fan J Wang B Traugh N Chen Q Liu JS. TIMER: A Web Server for Comprehensive Analysis of Tumor-Infiltrating Immune Cells. Cancer Res (2017) 77(21):e108–10. doi: 10.1158/0008-5472.CAN-17-0307 PMC6042652 29092952

[20] Finotello F Mayer C Plattner C Laschober G Rieder D Hackl H. Molecular and Pharmacological Modulators of the Tumor Immune Contexture Revealed by Deconvolution of RNA-Seq Data. Genome Med (2019) 11(1):34. doi: 10.1186/s13073-019-0638-6 31126321 PMC6534875

[21] Dienstmann R Villacampa G Sveen A Mason MJ Niedzwiecki D Nesbakken A. Relative Contribution of Clinicopathological Variables, Genomic Markers, Transcriptomic Subtyping and Microenvironment Features for Outcome Prediction in Stage II/III Colorectal Cancer. Ann Oncol (2019) 30(10):1622–9. doi: 10.1093/annonc/mdz287 PMC6857614 31504112

[22] Racle J de Jonge K Baumgaertner P Speiser DE Gfeller D. Simultaneous Enumeration of Cancer and Immune Cell Types From Bulk Tumor Gene Expression Data. Elife (2017) 6:e26476. doi: 10.7554/eLife.26476 29130882 PMC5718706

[23] Tamminga M Hiltermann TJN Schuuring E Timens W Fehrmann RS Groen HJ. Immune Microenvironment Composition in Non-Small Cell Lung Cancer and Its Association With Survival. Clin Transl Immunol (2020) 9(6):e1142. doi: 10.1002/cti2.1142 PMC7291326 32547744

[24] Chen B Khodadoust MS Liu CL Newman AM Alizadeh AA. Profiling Tumor Infiltrating Immune Cells With CIBERSORT. Methods Mol Biol (2018) 1711:243–59. doi: 10.1007/978-1-4939-7493-1_12 PMC5895181 29344893

[25] Charoentong P Finotello F Angelova M Mayer C Efremova M Rieder D. Pan-Cancer Immunogenomic Analyses Reveal Genotype-Immunophenotype Relationships and Predictors of Response to Checkpoint Blockade. Cell Rep (2017) 18(1):248–62. doi: 10.1016/j.celrep.2016.12.019 28052254

[26] Clyde D. Cancer Genomics: Keeping Score With Immunotherapy Response. Nat Rev Genet (2017) 18(3):146. doi: 10.1038/nrg.2017.2 28111471

[27] Chan TA Yarchoan M Jaffee E Swanton C Quezada SA Stenzinger A. Development of Tumor Mutation Burden as an Immunotherapy Biomarker: Utility for the Oncology Clinic. Ann Oncol (2019) 30(1):44–56. doi: 10.1093/annonc/mdy495 30395155 PMC6336005

[28] Chalmers ZR Connelly CF Fabrizio D Gay L Ali SM Ennis R. Analysis of 100,000 Human Cancer Genomes Reveals the Landscape of Tumor Mutational Burden. Genome Med (2017) 9(1):34. doi: 10.1186/s13073-017-0424-2 28420421 PMC5395719

[29] Samstein RM Lee CH Shoushtari AN Hellmann MD Shen R Janjigian YY. Tumor Mutational Load Predicts Survival After Immunotherapy Across Multiple Cancer Types. Nat Genet (2019) 51(2):202–6. doi: 10.1038/s41588-018-0312-8 PMC6365097 30643254

[30] van Rooij N van Buuren MM Philips D Velds A Toebes M Heemskerk B. Tumor Exome Analysis Reveals Neoantigen-Specific T-Cell Reactivity in an Ipilimumab-Responsive Melanoma. J Clin Oncol (2013) 31(32):e439–42. doi: 10.1200/JCO.2012.47.7521 PMC3836220 24043743

[31] Postow MA Sidlow R Hellmann MD. Immune-Related Adverse Events Associated With Immune Checkpoint Blockade. N Engl J Med (2018) 378(2):158–68. doi: 10.1056/NEJMra1703481 29320654

[32] Hegde PS Karanikas V Evers S. The Where, the When, and the How of Immune Monitoring for Cancer Immunotherapies in the Era of Checkpoint Inhibition. Clin Cancer Res (2016) 22(8):1865–74. doi: 10.1158/1078-0432.CCR-15-1507 27084740

[33] Whiteside TL Demaria S Rodriguez-Ruiz ME Zarour HM Melero I. Emerging Opportunities and Challenges in Cancer Immunotherapy. Clin Cancer Res (2016) 22(8):1845–55. doi: 10.1158/1078-0432.CCR-16-0049 PMC4943317 27084738

[34] Sautes-Fridman C Lawand M Giraldo NA Kaplon H Germain C Fridman WH. Tertiary Lymphoid Structures in Cancers: Prognostic Value, Regulation, and Manipulation for Therapeutic Intervention. Front Immunol (2016) 7:407. doi: 10.3389/fimmu.2016.00407 27752258 PMC5046074

[35] de Chaisemartin L Goc J Damotte D Validire P Magdeleinat P Alifano M. Characterization of Chemokines and Adhesion Molecules Associated With T Cell Presence in Tertiary Lymphoid Structures in Human Lung Cancer. Cancer Res (2011) 71(20):6391–9. doi: 10.1158/0008-5472.CAN-11-0952 21900403

[36] Helmink BA Reddy SM Gao J Zhang S Basar R Thakur R. B Cells and Tertiary Lymphoid Structures Promote Immunotherapy Response. Nature (2020) 577(7791):549–55. doi: 10.1038/s41586-019-1922-8 PMC8762581 31942075

[37] Kim JY Kronbichler A Eisenhut M Hong SH van der Vliet HJ Kang J. Tumor Mutational Burden and Efficacy of Immune Checkpoint Inhibitors: A Systematic Review and Meta-Analysis. Cancers (Basel) (2019) 11(11). doi: 10.3390/cancers11111798 PMC6895916 31731749

[38] Rizvi H Sanchez-Vega F La K Chatila W Jonsson P Halpenny D. Molecular Determinants of Response to Anti-Programmed Cell Death (PD)-1 and Anti-Programmed Death-Ligand 1 (PD-L1) Blockade in Patients With Non-Small-Cell Lung Cancer Profiled With Targeted Next-Generation Sequencing. J Clin Oncol (2018) 36(7):633–41. doi: 10.1200/JCO.2017.75.3384 PMC6075848 29337640

[39] Xie Z Li X Lun Y He Y Wu S Wang S. Papillary Thyroid Carcinoma With a High Tumor Mutation Burden Has a Poor Prognosis. Int Immunopharmacol (2020) 89(Pt B):107090. doi: 10.1016/j.intimp.2020.107090 33091816

[40] Li H Liu Y Liu J Sun Y Wu J Xiong Z. Assessment of ADCY9 Polymorphisms and Colorectal Cancer Risk in the Chinese Han Population. J Gene Med (2021) 23(2):e3298. doi: 10.1002/jgm.3298 33232543

[41] Yi H Wang K Jin JF Jin H Yang L Zou Y. Elevated Adenylyl Cyclase 9 Expression Is a Potential Prognostic Biomarker for Patients With Colon Cancer. Med Sci Monit (2018) 24:19–25. doi: 10.12659/msm.906002 29292367 PMC5759510

[42] Sun J Zhou C Ma Q Chen W Atyah M Yin Y. High GCLC Level in Tumor Tissues Is Associated With Poor Prognosis of Hepatocellular Carcinoma After Curative Resection. J Cancer (2019) 10(15):3333–43. doi: 10.7150/jca.29769 PMC6603424 31293636

[43] Kim AD Zhang R Han X Kang KA Piao MJ Maeng YH. Involvement of Glutathione and Glutathione Metabolizing Enzymes in Human Colorectal Cancer Cell Lines and Tissues. Mol Med Rep (2015) 12(3):4314–9. doi: 10.3892/mmr.2015.3902 26059756

[44] Li M Zhang Z Yuan J Zhang Y Jin X. Altered Glutamate Cysteine Ligase Expression and Activity in Renal Cell Carcinoma. BioMed Rep (2014) 2(6):831–4. doi: 10.3892/br.2014.359 PMC4179690 25279154

[45] Kuang Q Liang Y Zhuo Y Cai Z Jiang F Xie J. The ALDOA Metabolism Pathway as a Potential Target for Regulation of Prostate Cancer Proliferation. Onco Targets Ther (2021) 14:3353–66. doi: 10.2147/OTT.S290284 PMC8163754 34079281

[46] Shi S Ji S Qin Y Xu J Zhang B Xu W. Metabolic Tumor Burden is Associated With Major Oncogenomic Alterations and Serum Tumor Markers in Patients With Resected Pancreatic Cancer. Cancer Lett (2015) 360(2):227–33. doi: 10.1016/j.canlet.2015.02.014 25687883

[47] Long F Cai X Luo W Chen L Li K. Role of Aldolase A in Osteosarcoma Progression and Metastasis: *In Vitro* and *In Vivo* Evidence. Oncol Rep (2014) 32(5):2031–7. doi: 10.3892/or.2014.3473 25215901

[48] Du S Guan Z Hao L Song Y Wang L Gong L. Fructose-Bisphosphate Aldolase a Is a Potential Metastasis-Associated Marker of Lung Squamous Cell Carcinoma and Promotes Lung Cell Tumorigenesis and Migration. PloS One (2014) 9(1):e85804. doi: 10.1371/journal.pone.0085804 24465716 PMC3900443

[49] Lee D Xu IM Chiu DK Leibold J Tse AP Bao MH. Induction of Oxidative Stress Through Inhibition of Thioredoxin Reductase 1 Is an Effective Therapeutic Approach for Hepatocellular Carcinoma. Hepatology (2019) 69(4):1768–86. doi: 10.1002/hep.30467 PMC8690574 30561826

[50] Stafford WC Peng X Olofsson MH Zhang X Luci DK Lu L. Irreversible Inhibition of Cytosolic Thioredoxin Reductase 1 as a Mechanistic Basis for Anticancer Therapy. Sci Transl Med (2018) 10(428). doi: 10.1126/scitranslmed.aaf7444 PMC7059553 29444979

[51] van Kuilenburg AB Meinsma R. The Pivotal Role of Uridine-Cytidine Kinases in Pyrimidine Metabolism and Activation of Cytotoxic Nucleoside Analogues in Neuroblastoma. Biochim Biophys Acta (2016) 1862(9):1504–12. doi: 10.1016/j.bbadis.2016.05.012 27239701

[52] Yu S Li X Guo X Zhang H Qin R Wang M. UCK2 Upregulation Might Serve as an Indicator of Unfavorable Prognosis of Hepatocellular Carcinoma. IUBMB Life (2019) 71(1):105–12. doi: 10.1002/iub.1941 30304569

[53] Wu Y Jamal M Xie T Sun J Song T Yin Q. Uridine-Cytidine Kinase 2 (UCK2): A Potential Diagnostic and Prognostic Biomarker for Lung Cancer. Cancer Sci (2019) 110(9):2734–47. doi: 10.1111/cas.14125 PMC6726693 31278886

[54] Shaw RJ. Glucose Metabolism and Cancer. Curr Opin Cell Biol (2006) 18(6):598–608. doi: 10.1016/j.ceb.2006.10.005 17046224

[55] Gatenby RA Gillies RJ. Why do Cancers Have High Aerobic Glycolysis? Nat Rev Cancer (2004) 4(11):891–9. doi: 10.1038/nrc1478 15516961

[56] Shen J Jin Z Lv H Jin K Jonas K Zhu C. PFKP is Highly Expressed in Lung Cancer and Regulates Glucose Metabolism. Cell Oncol (Dordr) (2020) 43(4):617–29. doi: 10.1007/s13402-020-00508-6 PMC12990724 32219704

[57] Koguchi T Tanikawa C Mori J Kojima Y Matsuda K. Regulation of Myo-Inositol Biosynthesis by P53-ISYNA1 Pathway. Int J Oncol (2016) 48(6):2415–24. doi: 10.3892/ijo.2016.3456 27035231

[58] Guo X Li HH Hu J Duan YX Ren WG Guo Q. ISYNA1 Is Overexpressed in Bladder Carcinoma and Regulates Cell Proliferation and Apoptosis. Biochem Biophys Res Commun (2019) 519(2):246–52. doi: 10.1016/j.bbrc.2019.08.129 31495492

[59] Zenger S Ek-Rylander B Andersson G. Biogenesis of Tartrate-Resistant Acid Phosphatase Isoforms 5a and 5b in Stably Transfected MDA-MB-231 Breast Cancer Epithelial Cells. Biochim Biophys Acta (2010) 1803(5):598–607. doi: 10.1016/j.bbamcr.2010.01.021 20149826

[60] Gao YL Liu MR Yang SX Dong YJ Tan XF. Prognostic Significance of ACP5 Expression in Patients With Lung Adenocarcinoma. Clin Respir J (2018) 12(3):1100–5. doi: 10.1111/crj.12637 28398694

